# Oncologic Safety of Skin-Sparing and Nipple-Sparing Mastectomy: A Discussion and Review of the Literature

**DOI:** 10.1155/2012/921821

**Published:** 2012-07-17

**Authors:** Christopher Tokin, Anna Weiss, Jessica Wang-Rodriguez, Sarah L. Blair

**Affiliations:** ^1^Department of Surgery, University of San Diego, San Diego, CA 92103, USA; ^2^Department of Pathology, University of San Diego, San Diego, CA 92103, USA

## Abstract

Breast conservation therapy has been the cornerstone of the surgical treatment of breast cancer for the last 20 years; however, recently, the use of mastectomy has been increasing. Mastectomy is one of the most frequently performed breast operations, and with novel surgical techniques, preservation of the skin envelope and/or the nipple-areolar complex is commonly performed. The goal of this paper is to review the literature on skin-sparing mastectomy and nipple-sparing mastectomy and to evaluate the oncologic safety of these techniques. In addition, this paper will discuss the oncologic importance of margin status and type of mastectomy as it pertains to risk of local recurrence and relative need for adjuvant therapy.

## 1. Introduction

Since the advent of Halsted's radical mastectomy in the 1800's, the surgical treatment of breast cancer has become increasingly refined. Today, the radical mastectomy is rarely performed, however, with breast cancer affecting nearly one in eight women [1], it remains an important part of breast cancer treatment, especially for more advanced or locally aggressive tumors.

Since the increasing trend towards breast conservation therapy (BCT), the surgical literature has focused on the predictors of locoregional recurrence (LR) after BCT. However, there has been a recent swing on the pendulum back towards a higher rate of mastectomy [2], utilizing new surgical techniques where the skin and/or nipple-areolar complex (NAC) can be preserved. These techniques are being used to improve postoperative cosmesis, and it is important to understand how these procedures differ from the basic modified-radical mastectomy (MRM), and how important particular demographic, technical, and tumor-specific factors are at predicting LR and oncologic safety with these various techniques. 

## 2. The Skin-Sparing Mastectomy

 The modified radical mastectomy (MRM), or traditional non-skin-sparing mastectomy (NSSM), most commonly performed today, was described by Madden in 1965. This procedure involves removal of all breast tissue, while preserving both pectoralis muscles, and it is commonly accompanied by dissection of level I and II axillary lymph nodes if indicated [3]. A locoregional recurrence (LR) rate of roughly 10% at 5–8 years is deemed acceptable by many authors [4–7], with most LR occurring within the first five postoperative years [5].

The skin-sparing mastectomy (SSM) was first described in 1991 by Toth and Lappert [33] as an effort to maximize skin preservation to improve cosmetic outcome and facilitate reconstruction. It typically entails removal of the entire breast and nipple-areola complex (NAC) while preserving the skin envelope and the natural inframammary fold [34]. The dissection is carried out in the same plane as the NSSM and an effort to remove an equivalent amount of breast tissue as in NSSM should be made [35]. The traditional SSM also involves excision of the skin overlying superficial tumors as well as previous biopsy entry sites to decrease chances of LR, however, this is not routinely performed by all surgeons [36]. 

Naturally, leaving behind additional tissue is of concern when oncologic safety is imperative. The most common site for LR after conventional mastectomy is within the skin overlying the chest wall and LR often portends a poor prognosis [37–39]. SSM is less ablative than NSSM, leaving behind superior and inferior skin flaps to preserve the natural skin envelope, however, the technique still requires the surgeon to remove as much breast tissue as possible, with dissection carried out above the superficial fascia, leaving behind only epidermis, dermis, and a small amount of subcutaneous fat. Diminished exposure and a larger area of increased residual skin make the procedure more technically demanding. Recent reports have characterized the histological characteristics of skin flap specimens. Identifying residual breast tissue or positive superficial margins carries with it concern for oncologic safety and equivalence at maintaining local control [40, 41]. 

Torresan et al. looked at the amount of residual breast tissue after SSM by histologically analyzing skin flap specimens, and they found 59.5% of specimens contained residual breast tissue, and 9.5% of skin flaps harbored residual disease [42, 43]. They also concluded that skin flaps >5 mm were associated with the presence of residual disease. 

Ho et al. [44] found 23% of analyzed skin flaps were involved with residual tumor, with the majority of involved skin flaps located directly over the tumor, and significantly associated with tumor size and the presence of skin tethering. Cao et al. histologically analyzed additional skin margins (ASM) taken at the time of SSM and found that 38% of 168 SSM's had a positive superficial specimen margin. A thicker ASM had residual breast tissue in 53% of cases, and it was an independent predictor of residual disease [45]. 

It is imperative that the oncologic breast surgeon strives for clear margins, and yet few studies have reported on margin status after SSM and the literature is varied. Despite numerous studies previously mentioned, showing no statistical difference between NSSM and SSM in terms of LR, SSM has been shown to be an independent predictor of close or positive margins [42, 43, 45, 46]. Horiguchi et al. defined close margin as within 5 mm and did find margin status significant on multivariate analysis [47]. Smaller specimen weight, smaller skin area, a lower skin/SSM surface ratio, an extensive insitu component, hormone receptor status, younger patient age, multiple ipsilateral tumors, palpable cancers, and tumor location (specifically the upper inner quadrant) have all been shown to be independent risk factors for positive margins in SSM (see Table 2) [45, 46, 48].

Despite these findings showing relatively high rates of residual breast tissue with or without involved superficial margins, numerous studies over the past two decades have determined that SSM is an oncologically safe procedure with no significant difference in LR than NSSM [8, 12, 13, 40, 49–53]. The LR after NSSM in tumors up to 4 cm was shown to be 10% after 20 years of followup [54]. An extensive review of the literature has shown that SSM recurrence rates range from 0–7% [11, 50]. These studies include both prospective and retrospective design, and they are difficult to compare as they vary largely in patient sample size, stage of disease, tumor characteristics, adjuvant chemotherapy or radiation therapy, use and type of immediate or delayed reconstruction, and followup time. Several meta-analyses have concluded that SSM is an oncologically safe procedure, at least for early stage and small tumor size [35, 55].

Overall, as would be expected, LR after SSM is lower for smaller and low stage tumors with less invasive characteristics. LR after mastectomy for DCIS in most series is between 1–3% [56–59]. Similarly, Slavin et al. [11] had no recurrences over 45 months for 26 cases of SSM for DCIS. The series by Carlson [49] included 175 cases of DCIS, and on subset analysis, after 65 months of followup, there was only one LR. These findings are supported by several other studies, summarized in Table 1. 

When T1 and T2 tumors were looked at specifically, Newman et al. [40] reported a 6.2% recurrence after SSM and immediate reconstruction (IR) with a mean followup time of 26 months. This was in agreement with Kroll and Khoo [50] who reported a 7% LR with SSM and IR, as compared to a 7.5% NSSM. The largest series by Carlson et al. mentioned previously involved 539 patients, with a mean followup of 65 months, and found that tumor size, nodal status, and LVI were all significant predictors of recurrence, with LR of 3, 10, and 11% for T1, T2, and T3 tumors, respectively [49]. Medina Franco et al. [52] also reported that tumor size, stage, lymph node involvement, and poor tumor differentiation were risk factors for LR, and they reported that after SSM, LR was 4.5% in 176 cancers with a median followup of 73 months. Spiegel and Butler [12] followed patients for 9.8 years, and they reported an LR of 5.6% in 117 invasive cancers after SSM. 

Studies of SSM in high-risk patients, particularly those with more advanced stage, have also been selectively performed, albeit in fairly small patient populations, however, have shown promising results, with recurrence rates between 2.6–4.6% [13–15]. These results are summarized in Table 3.

The majority of mastectomy patients do not require adjuvant radiotherapy, however, those with more than three positive regional nodes or larger tumors are often offered radiotherapy in addition to mastectomy [60]. SSM is most often studied as an alternative to NSSM, however, with preservation of the skin envelope, unlike NSSM, SSM mandates a reconstructive procedure. Previously, SSM had been avoided in situations that require adjuvant radiotherapy because of the risk of complications associated with radiotherapy and immediate reconstruction (IR), with rates that vary from 5–16% [61]. While the different types of reconstructive procedures performed with SSM are beyond the scope of this review, complications have been diminished by use of a temporary tissue expander placed underneath the pectoralis major at the time of IR. At a second operation, the expander is replaced with a permanent implant or delayed reconstruction using a myocutaneous flap is performed after the area has been irradiated, protecting the flap from damage [61]. While capsular contraction has been reported as a common response to adjuvant radiation after implant or expander placement [62, 63], Hughes et al. found that skin-sparing mastectomy followed by irradiation did increase the rate of reoperation and did not lead to significant increase in complications or capsular contracture rates [62]. Furthermore, despite having adjuvant radiotherapy, patient satisfaction scores regarding aesthetic outcome remain high. [63] 

SSM is an oncologically safe procedure with LR rates comparable to NSSM for small and low-grade tumors, and the literature suggests this may also be true for tumors of higher grade and stage. Margins after SSM are as important, if not more important than after NSSM because of the decreased operative exposure and the technique to make adequately thin skin flaps make obtaining clear margins more difficult. The surgeon should be aware of risk factors for positive margins after SSM in order to be vigilant about an adequate cancer extirpation. Because of the preservation of the skin envelope, SSM does require reconstruction be performed at the time of the mastectomy. While adjuvant radiotherapy in the setting of IR after SSM is not preferred given higher rates of complications such as fat necrosis or capsular contracture, techniques such as IR with tissue expander placement can diminish complication rates and maintain the improved aesthetic outcomes that SSM can offer over NSSM. 

## 3. The Nipple-Sparing Mastectomy

Nipple-sparing mastectomy (NSM) is similar to SSM but spares the nipple-areolar complex (NAC), mandating removal of nipple-areolar (NA) ducts [21, 64] and leaving only the epidermis and dermis at the NA behind. Recommendations are that skin flaps in NSM should only be 2–3 mm in thickness at the NAC [21], with the technique facilitated by nipple eversion during dissection, and use of sharp dissection instead of electrocautery to limit thermal injury and increase NA preservation rates  [64]. 

The NA ducts are commonly sent as separate specimens. Some surgeons send this tissue for frozen section examination of the NAC for residual cancer with studies advocating removal of the entire NAC (conversion to SSM) if frozen section is positive for disease [28, 64, 65]. Other groups will wait for permanent section and return to the operating room for ultimate removal of the NAC if final pathology is positive. Fortunately, residual disease in NAC tissue is rare in carefully selected patients [24]. Some groups recommend use of intraoperative radiotherapy when the NSM technique is employed  [19]. Similar to SSM, preservation of the NAC and skin envelope then mandates immediate reconstruction [26, 66]. 

Multiple techniques and approaches have been described in an effort to prevent NA necrosis, which can be a complication of NSM given the close dissection under the NAC. Outcomes of these various techniques can be found in Table 3. 

Several authors have shown that certain incisions are associated with a decreased risk of necrosis, particularly if the surgeon ensures that the incision does not extend across the whole circumference of the NAC, loss of the nipple is less likely  [31]. Stolier et al. performed 82 NSMs without NA necrosis, and advocated a six-o'clock radial incision, or a lateral incision if excising a biopsy or BCT scar  [25]. They also stressed the importance of lighting, use of headlamps, blended current cautery used only for pinpoint homeostasis, and the utility of bipolar dissecting scissors. Other authors also endorse the use of radial or lateral incisions,  [21, 22]  noting that medial incisions seemed to compromise blood flow  [21]. Paepke et al. reported only a 1% NA loss with a periareolar incision, [28], however, Regolo et al. reported a 60% NA loss with periareolar incision, [23] which they abandoned in favor of a lateral incision. In summary, since there is no agreement on optimal approach, surgeons should be familiar with the literature and employ an approach they are familiar with for optimal outcomes.

 It is generally accepted that NSM provides better cosmetic results than MRM, however, there is little literature comparing the two procedures. Outcomes of immediate breast reconstruction are improved with SSM because the natural skin envelope is preserved; it is natural to extend this principle to the preservation of the nipple [67]. In a study by Gerber et al., [17] patients and surgeons evaluated aesthetic results of SSM versus NSM after 12 months. Patients rated satisfaction with SSM and NSM similarly with the majority ranked aesthetic outcome as good or excellent. The surgeons, however, rated 74% of NSM excellent and 26% good, while rating only 59% of SSM excellent, 22% good, and 20% fair (*P* = 0.001). Another study [68] focused on patient satisfaction with body image, sexuality, cosmetic results, and psychological adjustment. They found no difference in feelings of sexuality. However, those with NSM were more willing to see themselves or be seen naked, and had significantly lower ratings for feelings of mutilation. Patients who underwent NSM as compared to SSM reported significantly greater satisfaction with cosmetic results.

Secondary to improved cosmesis, NSM has gained popularity for patients requiring or choosing mastectomy. Therefore, the oncologic safety must be closely evaluated. Despite being commonly offered as an alternative to NSSM, indications for NSM have typically been identical to those for BCT [29, 35, 64]. NSM has been considered safe in women with small, peripherally located tumors, without multicentricity, or for prophylactic mastectomy [29]. Multiple prospective and retrospective studies have been done looking at LR in NSM to address oncologic safety, a summary of which can be found in Table 4. 

Three landmark studies that looked at the incidence of microscopic tumor involvement in the NAC had conflicting results. In 1999, Laronga et al. reported that 5.6% of NAC in SSM specimens were positive for occult tumor involvement [69], and they concluded that NAC involvement was not an indicator of increased LR or breast cancer specific survival. They did report that central tumor location, multicentricity, and positive lymph nodes did carry an increased risk of NA involvement. In 2001, Cense et al. reported that up to 58% of mastectomy specimens had NA involvement [70], which correlated with tumor size, distance from the NA complex (<4-5 cm), or positive lymph nodes. Since there was high NAC involvement, and because patients with mastectomy often forego adjuvant radiotherapy, they recommended that NSM carried with it unacceptable oncologic risk, and they recommended that patients should defer to BCT. However, in agreement with Laronga et al., in 2002, Simmons et al. reported 10.6% of mastectomy specimens and 6.7% of T1 and peripherally located tumors had NA involvement [71]. They studied the NAC separately and found only 0.9% of specimens and areolar involvement. In a retrospective study of 575 bilateral prophylactic mastectectomies for high-risk patients, Hartmann et al. described performing a subcutaneous mastectomy. In this procedure, which leaves more tissue under the NAC than NSM, they reported a low 1.2% LR, with only 0.2%LR involving the NAC with two years of followup [72]. 

 The literature reveals that the LR after NSM was between 0^56^–20^65^%, with studies varying widely in patient size, inclusion criteria, and followup. Benediktsson and Perbeck [73] offered NSM to patients who were poor BCT candidates, thus patients with large and multicentric tumors were included. They reported an LR of 20.8%, but attributed it to failure to treat with adjuvant radiation, and despite LR, 0% of patients had recurrence at the NAC with 13 years of followup. With PMRT added to NSM, they saw a statistically significant reduction in LR to 8.5%, suggesting that NSM may be an oncologically safe procedure in more advanced disease with the addition of PMRT. Petit et al. [19] and Sookhan et al. [26] endorsed use of breast magnetic resonance imaging (MRI) as a useful preoperative tool, as both reported 0% NAC LR, albeit with minimal followup periods of 19 months and 10.8 months respectively.

More recent reports advocate for the oncological safety of NSM. In 2009, Gerber et al. provided almost 10 years of extended followup data, and they found that NSM was oncologically safe, with only one NAC recurrence out of 112 NSMs performed, and no statistical significance between overall LR between NSM and MRM [74]. In 2012, Petit et al. reported that 10% of NA specimens were positive after frozen section, however, with the use of intraoperative radiotherapy, their long-term NAC recurrence was 1.18% [75]. 

There is evidence that NSM is oncologically safe if performed as a prophylactic mastectomy or in patients who would, otherwise, be candidates for BCT, however, as with SSM, the additional tissue left behind may be associated with an increase in LR over conventional mastectomy for more advanced tumors with more aggressive biology. While there is data supporting the safety of SSM for larger tumors and more advanced stages, there is less applied to NSM, and additional study, preferably prospective, should be performed. The literature regarding margins for NSM focuses on the margin at the NAC, but it is prudent to remember that superficial and deep margins apply as well, and this has not been sufficiently studied or addressed. 

## 4. Conclusion

There are many reasons that women may chose mastectomy over BCT, and some, even bilateral mastectomy over unilateral—better and more diverse reconstructive options, perceived risk or anxiety, requirements for surveillance and imaging, and genetic syndromes such as BRCA carriers. Breast cancer is a complex disease, and local recurrence and cancer-related death is likely multifactorial. Since a consensus on what defines close margins, or what type of adjuvant therapy should be applied to mastectomies of different types is lacking, researchers should continue with efforts on answering these questions. The goal for the oncologic surgeon should be complete cancer extirpation with negative margins, and regardless of type of mastectomy performed, more aggressive tumor biology in patients of younger age may warrant more aggressive treatment strategies. 

## Figures and Tables

**Table 1:** Local recurrence after skin-sparing mastectomy in stage DCIS.

Author	Study design	Number of Pts	LR	Followup (months)
Carlson et al. [8]	Retrospective	175	0.6%	65
Greenway et al. [9]	Retrospective	28	0%	49
Rubio et al. [10]	Retrospective	95	3%	44.4
Slavin et al. [11]	Retrospective	26	0%	45
Spiegel and Butler [12]	Retrospective	44	0%	117.6

**Table 2:** Local recurrence after skin-sparing mastectomy in stage IIB/III breast cancer.

Author	Study design	Number of Pts	LR	Followup (months)
Lim et al. [13]	Retrospective	87	4.6%	60
Foster et al. [14]	Prospective	25	4%	49.2
Downes et al. [15]	Retrospective	38	2.6%	52.9

**Table 3:** Rate of NAC loss following NSM.

Author	Year	Number of Pts	NA complex loss	Incision
Petit et al. [16]	2003	27	3.7% total, 7.4% partial	
Gerber et al. [17]	2003	112	0% total, 9.8% partial	
Crowe et al. [18]	2004	54	0% total, 6% partial	Medial led to all 3 losses (6%), then used lateral
Petit et al. [19]	2006	106	4.7% total, 10.4% partial	Ellipse overlying previous biopsy site
Caruso et al. [20]	2006	50	0.02% total	
Sacchini et al. [21]	2006	192	4.7% >2/3 area loss, 11.45% partial	Primarily PA^∗^ with lateral extension
Crowe et al. [22]	2008	149	0.67% total, 1.3% partial	Lateral
Regolo et al. [23]	2008	102	60% in first 32 (periareolar incision), 2.8% NAC complication rate thereafter	Primarily PA, then lateral
Wijayanayagam et al. [24]	2008	64	5% total, 16% partial	97% NA survival in radial, if PA > 1/3 NA circumference blood supply compromised
Stolier et al. [25]	2008	82	0%	6 o'clock radial
Sookhan et al. [26]	2008	18	0% total, 10% partial	Inframammary
Petit et al. [27]	2009	1001	3.5% total, 5.5% partial	Overlying previous biopsy
Paepke et al. [28]	2009	96	1%	Primarily PA, second inframammary
De Alcantara Filho et al. [29]	2011	353	19.5% partial, 3.3% required surgery	

**Table 4:** Local and nipple areolar complex recurrence after nipple-sparing mastectomy.

Author	Study design	Number of Pts	LR	NAC recurrence	Median followup (months)
Gerber et al. [17]	Prospective	112	5.4% NSM, 8.2% MRM (*P* = 0.6)	0.9%	59
Petit et al. [16]	Prospective	27	0.00%	0	6
Caruso et al. [20]	Prospective	50	2%	2%	66
Sacchini et al. [21]	Retrospective	192	3%	0	24.6
Petit et al. [19]	Prospective	106	1%	0	13
Benediktsson and Perbeck [73]	Prospective	216	20.8%	0	156
Regolo et al. [23]	Retrospective	102	0	0	16
Petit et al. [27]	Prospective	579	2.40%	0	19
Crowe et al. [22]	Prospective	149	1.30%	0	41
Sookhan et al. [26]	Retrospective	18	0	0	10.8
Voltura et al. [30]	Retrospective	51	5.9	0	18
Garwood et al. [31]	Prospective	102	0.6%	0	13
Gerber et al. [74]	Prospective	112^∗^	11.7% NSM, 10.4% SSM, 11.5% MRM (*P* = 0.974)	0.9%	101
Garcia-Etienne et al. [32]	Retrospective	42 NSM	0	0	10
Paepke et al. [28]	Retrospective	96	2%	0	34
De Alcantara Filho et al. [29]	Prospective	353	0	0	10.4
Petit et al. [75]	Prospective	934	4%	1.18%	50
